# 

**Published:** 1876-03

**Authors:** 


### THE SOUTHERN MEDICAL RECORD:

PUBLISHED MONTHLY AT

ATLANTA, GEORGIA.

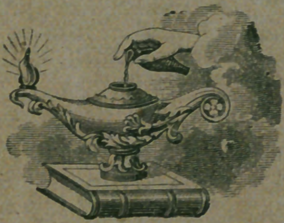

LOCAL EDITORS:

THOS. S. POWELL, M.D. R. C. WORD, M.D

W. T. GOLDSMITH, M.D. H. B. LEE, M.D.

ASSOCIATE EDITORS:

T. CURTIS SMITH, M.D.........Middleport, Ohio.
SWAN M. BURNETT, M.D .......Knoxville, Tenn.
ALBAN S. PAYNE, M.D.... ......Markham's, Va.
A. R. KILPATRICK, M D..........Navasota, Texas.
A. A. LYON, M.D.... ............Shreveport, La.
G. A. BAXTER, M.D ..............Chattanooga, Tenn.
J. B. MATTISON, M.D............Brooklyn, N. Y.

*"QUICQUID PRÆCIPIES ESTO BREVIS."*

ATLANTA, GA.:

JAS. P. HARRISON & CO., PRINTERS AND BINDERS.

1876.

